# Activation of TGF-β signaling in an aortic aneurysm in a patient with Loeys-Dietz syndrome caused by a novel loss-of-function variant of *TGFBR1*

**DOI:** 10.1038/s41439-019-0038-x

**Published:** 2019-01-18

**Authors:** Hironori Hara, Norifumi Takeda, Takayuki Fujiwara, Hiroki Yagi, Sonoko Maemura, Tsubasa Kanaya, Kan Nawata, Hiroyuki Morita, Issei Komuro

**Affiliations:** 1 Department of Cardiovascular Medicine, The University of Tokyo Hospital, 7-3-1 Hongo, Bunkyo-ku, Tokyo 113-8655 Japan; 2 Department of Cardiac Surgery, The University of Tokyo Hospital, 7-3-1 Hongo, Bunkyo-ku, Tokyo 113-8655 Japan

**Keywords:** Cardiovascular diseases, Clinical genetics

## Abstract

Loeys–Dietz syndrome (LDS) is caused by variants of transforming growth factor-β (TGF-β)-related genes and is characterized by aortic aneurysm and dissection. We report an LDS patient with a de novo missense variant of *TGFBR1* [c.1126A>G, p.(Lys376Glu)] in which active TGF-β signaling was observed in the aorta, despite the in vitro demonstration that the loss-of-function mutation lies within the serine/threonine kinase domain. The mechanism underlying this TGF-β paradox in LDS aortopathy should be further investigated.

Loeys–Dietz syndrome (LDS) is an autosomal dominant genetic connective tissue disorder that is caused by pathogenic variants of genes that encode components of the transforming growth factor-β (TGF-β) signaling pathway. *TGFBR1* and *TGFBR2*, which encode serine/threonine protein kinase (STK) receptors, are examples of such causative genes^1,2^. LDS patients have several features that overlap symptoms seen in Marfan syndrome (MFS) patients, including an increased prevalence of aortic aneurysm and dissection. However, LDS patients do not develop ectopia lentis, which is a hallmark feature of MFS patients, and are further characterized by the diagnostic clinical triad of arterial tortuosity and aneurysms, hypertelorism, and a bifid uvula. The TGF-β signaling pathway in the aortic wall is upregulated in LDS patients, although the causative variants of *TGFBR1* and *TGFBR2* are missense variants predicted to reduce their STK activities^3^. To understand the crucial roles played by TGF-β signaling in LDS patients, in vitro and in vivo functional characterization of the genetic variants responsible for LDS is important. Here we report a case of an LDS patient with a de novo loss-of-function variant in the STK domain of *TGFBR1* (c.1126A>G; NG_007461.1), in which a paradoxical upregulation of TGF-β signaling was observed in the resected aortic tissue.

The patient was a Japanese man with no apparent relevant family history of thoracic aortic aneurysm and/or dissection. It was thought that he suffered from an MFS-related disorder because of his tall stature, thin body habitus (height, 182 cm; weight, 43.6 kg), and scoliosis at the age of 15 years. Echocardiography revealed an enlargement of the sinus of Valsalva (36 mm; aortic root *Z*-score^4^, 5.87), for which he had been followed up at a local hospital. At the age of 27 years, his aortic root diameter was 41 mm (188 cm; 50 kg; *Z*-score, 5.17), and he was referred to the Marfan clinic at the University of Tokyo Hospital. He had a positive wrist and thumb sign and presented with pectus excavatum, a hindfoot deformity, lumbosacral dural ectasia, protrusio acetabuli, kyphoscoliosis, facial features (dolichocephaly, downslanting palpebral fissures, and malar hypoplasia), skin striae, high myopia, and mitral valve prolapse (15 points, according to the 2010 revised Ghent nosology^5^). Furthermore, he had a bifid uvula, hypertelorism, tortuous cerebral arteries, and cervical spine instability. At the age of 31, his aortic root diameter was 48 mm (*Z*-score, 8.08), and his aneurysm was treated using the David valve-sparing root replacement procedure. Extended histological examinations revealed elastin degradation, cystic medial necrosis, and increased SMAD2 phosphorylation, indicating active TGF-β signaling in the aortic wall (Fig. 1a, b).

**Fig. 1:**
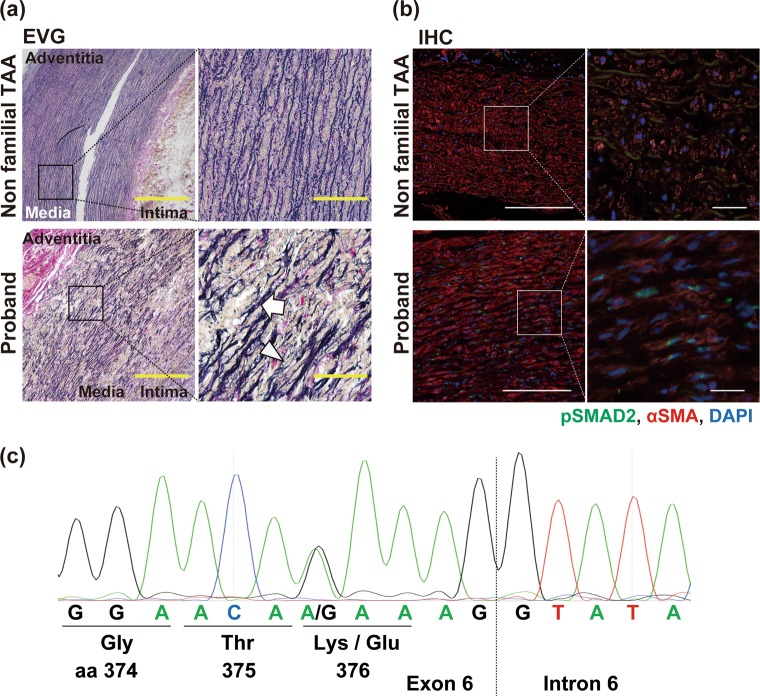
Histological and *TGFBR1* genomic analyses. **a**, **b** Histological analyses of surgically dissected aortic tissue samples from the proband with LDS and from a non-familial thoracic aortic aneurysm (TAA) in a 70-year-old patient. **a** Elastica van Gieson (EVG) staining revealed severely disorganized and fragmented elastic fibers (arrowhead) in the proband’s aortic media with cyst-like lesions (cystic medial necrosis, arrow). Scale bars: 500 µm; 100 µm in the inset. **b** Immunohistochemical (IHC) staining against phosphorylated SMAD2 (Cell Signaling, Danvers, MA, USA) and smooth muscle alpha-actin (Sigma, St. Louis, MO, USA). Scale bars: 200 µm; 25 µm in the inset. **c** Genomic DNA sequencing revealed a heterozygous single-base substitution (c.1126A>G) in the proband

Genetic analyses of thoracic aortic aneurysm-related genes (*FBN1, TGFBR1, TGFBR2, TGFB2, TGFB3, SMAD3, ACTA2*, and *MYH11*)^6,7^ were conducted. A novel missense variant was identified within exon 6 of *TGFBR1* [c.1126A>G, p.(Lys376Glu); Fig. 1c], and this variant was located in the evolutionarily well-conserved STK domain (data not shown). To examine the impact of the STK domain variant on TGFBR1 function, an in vitro functional assay was performed using wild-type and variant TGFBR1 expression vectors. A plasmid encoding human TGFBR1 with a C-terminal HA-tag (pCMV5-TBR1-HA, abbreviated as WT) was generated from a plasmid encoding a genetically altered human TGFBR1 mutant with intrinsic constitutive activity and a C-terminal HA-tag (pCMV5-TBR1-T204D-HA, abbreviated as CA; Addgene #19162, a gift from Joan Massague) by site-directed mutagenesis. Plasmids with the LDS missense variant (pCMV5-TBR1-LDS-HA and pCMV5-TBR1-T204D4-LDS-HA, abbreviated as constructs LDS and LDS-CA, respectively) were generated from the WT and CA constructs, respectively, through site-directed mutagenesis. The plasmid-encoded wild-type and variant TGFBR1 proteins were expressed at uniformly high levels in HEK293T cells (Fig. 2a), and there was low endogenous TGFBR1 expression. Using these constructs, luciferase assays were performed to assess the activity of the SMAD-responsive Smad-binding element (SBE) reporter in HEK293T cells co-transfected with the TGFBR1 expression vectors and either the pGL4.48 (luc2P/SBE/Hygro) vector (Promega, Madison, WI, USA) or the pRL-SV40 control vector (Promega, Madison, WI, USA)^7^. After treatment with recombinant human TGF-β1 (Wako, Osaka, Japan), the increased SBE luciferase activity seen in the WT-transfected HEK293T cells was abolished in the LDS-transfected cells (Fig. 2b). The high constitutive STK activity observed in the CA-transfected HEK293T cells was significantly inhibited in the LDS-CA-transfected cells (Fig. 2c). These results indicate that, at least in vitro, the Lys376Glu variant is a loss-of-function mutation. From these results, we concluded that this variant could be classified as likely pathogenic without confirmation of paternity and maternity based on the classification guidelines of the American College of Medical Genetics and Genomics**−**Association for Molecular Pathology^8^. The patient was finally diagnosed with LDS based on a constellation of clinical features and the results of the genetic analysis. The *TGFBR1* variant data were submitted to the Leiden Open Variant Database (www.LOVD.nl/TGFBR1; Individual ID: #00151836).

**Fig. 2:**
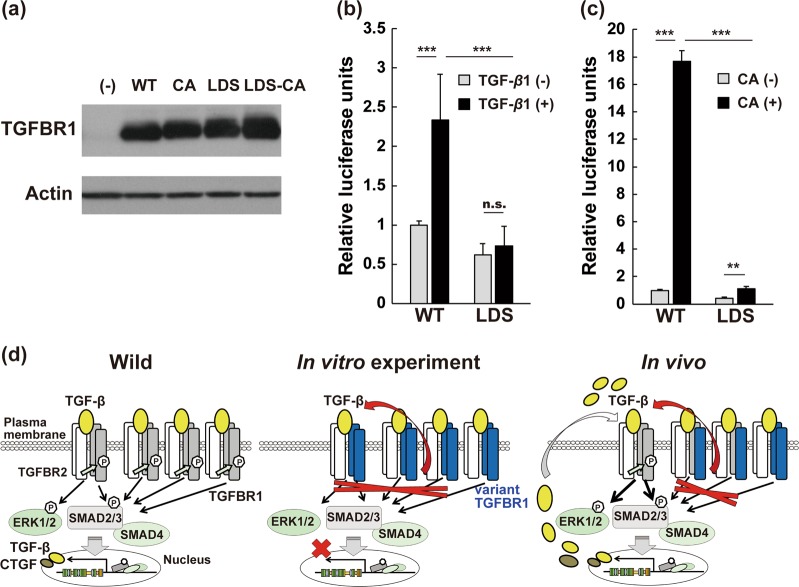
Functional assay of the Lys376Glu variant of the *TGFBR1* gene. **a** Western blot analysis for TGFBR1 expression from the newly generated TGFBR1 constructs. **b**, **c** SBE-firefly luciferase activity in HEK293T cells. **b** HEK293T cells transfected with the indicated constructs were cultured for 36 h; after 4 h of starvation in FCS-free DMEM, the cells were exposed to recombinant human TGF-β1 (Wako, Osaka, Japan) at 5 ng/ml for 8 h. *n* = 11. **c** HEK293T cells were transfected with the indicated constructs and harvested 48 h after transfection. *n* = 11 or 12. CA, constitutively active form. Data are shown as the mean ± SD. The statistical significance of the differences between the means was evaluated using one-way analysis of variance followed by the Tukey–Kramer test. n.s. *p* > 0.05, **p < 0.01, ***p <0.001. **d** Dysregulated TGF-β signaling elicited by the LDS-causing *TGFBR1* mutation. *Left*: The TGFBR1 (gray receptor) and TGFBR2 (white receptor) STKs acts as homodimers on the cell membrane in a normal aorta. TGF-β ligands binds to TGFBR2, inducing its dimerization and enabling the TGFBR2 homodimer to form a stable hetero-tetrameric complex with the TGFBR1 homodimer. This process leads to subsequent activation of SMAD2 and SMAD3, and SMAD-independent pathways (including ERK). *Middle*: An in vitro functional assay using the overexpressed variant TGFBR1 (blue receptor). The pathogenic missense mutation in the STK domain leads to a loss-of-function phenotype, as demonstrated in the present study (**b**, **c**). *Right*: In the impaired LDS aortic wall, which carries the loss-of-function variant, oversecreted TGF-β can be transmitted through the remaining wild-type TGFBR1 homodimers, which is believed to promote aortic aneurysm formation. White receptors, wild-type TGFBR2; gray receptors, wild-type TGFBR1; blue receptors, variant TGFBR1

Here, we report a Japanese sporadic LDS patient characterized by aortic aneurysm and a heterozygous missense *TGFBR1* variant (c.1126 A>G). In LDS patients, most of the *TGFBR1/2* variants are missense mutations that have been verified and/or predicted to disrupt the activity of the wild-type proteins upon in vitro overexpression. As expected, overexpression of the missense variant *TGFBR1* (c.1126 A>G) showed a loss-of-function phenotype in vitro; however, a paradoxical increase in TGF-β activity was observed in the aortic wall. The mechanism through which loss-of-function *TGFBR1/2* variants cause the paradoxical in vivo activation of TGF-β signaling in LDS aortopathy remains unknown^1,7,9^. Heterozygous *Tgfbr1* and *Tgfbr2* knockout mice do not develop LDS phenotypes; however, complete deletion of *Tgfbr2* in postnatal smooth muscles (*Myh11-CreERT2*;*Tgfbr2*^*f/f*^), along with the resulting decrease in phospho-SMAD2 expression, induces rapidly progressing aortic aneurysms and dissections^10,11^. These data indicate that basal TGF-β signaling in smooth muscles promotes postnatal aortic wall homeostasis and hinders aortic dilatation. Conversely, TGF-β signaling in LDS-associated aortic aneurysms is upregulated, as reported both in LDS patients and *Tgfbr1*^*M318R/+*^ and *Tgfbr2*^*G357W/+*^ LDS knock-in mice, despite their loss-of-function phenotypes shown by in vitro experiments^1,7,11^. Based on the following points, we (and others) speculate that augmented TGF-β signaling via the remaining homodimers of each TGFBR protein plays active roles in disease progression in LDS patients:^9,12^ (1) Expression of TGF-β ligands was increased in the impaired aortas of *Tgfbr1*^*M318R/+*^ and *Tgfbr2*^*G357W/+*^ mice;^11^ (2) TGFBR1 and TGFBR2 act as homodimers on the plasma membrane, which allows TGF-β signaling to be transmitted through the wild-type/wild-type homodimers in vivo in LDS; (3) increased phosphorylation of SMAD2 was observed in in vitro-cultured fibroblasts from LDS patients following TGF-β stimulation^1^. Furthermore, cultured aortic vascular smooth muscle cells from *Tgfbr2*^*G357W/+*^ mice showed normal steady-state SMAD2 phosphorylation levels and in response to stimulation with 10 ng/ml of TGF-β1, but showed reduced activation capacity in response to stimulation with 1 ng/ml of TGF-β1^11^, suggesting that excessive activation of TGF-β signaling plays a pivotal role in the pathogenesis of LDS aortopathy (Fig. 2d). On the other hand, we also suspect that the complete ablation of genes encoding TGF-β signaling molecules in *Myh11-CreERT2*;*Tgfbr2*^*f/f*^ mice^10^ and in homozygous *Smad3* knockout mice (*Smad3*^*−/−*^)^13^ may cause aortic aneurysm via other mechanisms different from those underlying LDS, as the canonical SMAD target genes are generally not upregulated. Angiotensin II receptor signaling is also activated in LDS aortic walls, and the type I receptor blocker losartan inhibits SMAD2 phosphorylation and improves aortic root dilatation in *Tgfbr2*^*G357W/+*^ LDS knock-in mice; thus, further studies on the relationships between the TGF-β and angiotensin II receptor signaling pathways in LDS patients are also required^11,14^.

In conclusion, we have described a patient with a sporadic case of LDS due to a novel *TGFBR1* variant (c.1126A>G) who presented with Marfan-like habitus, a bifid uvula, hypertelorism, and an aortic aneurysm. The loss of STK activity in the missense variant protein caused a paradoxical increase in aortic TGF-β signaling; however, the precise mechanism remains to be elucidated. We suspect that secondarily activated TGF-β signaling via wild-type TGFBR1 homodimers underlies the discrepancy between the in vitro and in vivo STK activities. Further investigation is crucial to clarify the molecular mechanism underlying the “TGF-β paradox” in LDS aortopathy.

## Data Availability

The relevant data from this Data Report are hosted at the Human Genome Variation Database at 10.6084/m9./share.hgv.2516

## References

[1] Loeys BL (2005). A syndrome of altered cardiovascular, craniofacial, neurocognitive and skeletal development caused by mutations in TGFBR1 or TGFBR2. Nat. Genet..

[2] Loeys BL (2006). Aneurysm syndromes caused by mutations in the TGF-β receptor. N. Engl. J. Med..

[3] Cardoso S, Robertson SP, Daniel PB (2012). TGFBR1 mutations associated with Loeys-Dietz syndrome are inactivating. J. Recept. Signal. Transduct..

[4] Roman MJ, Devereux RB, Kramer-Fox R, O’Loughlin J (1989). Two-dimensional echocardiographic aortic root dimensions in normal children and adults. Am. J. Cardiol..

[5] Loeys BL (2010). The revised Ghent nosology for the Marfan syndrome. J. Med. Genet..

[6] Takeda N (2015). A deleterious MYH11 mutation causing familial thoracic aortic dissection. Hum. Genome Var..

[7] Fujiwara T (2018). Distinct variants affecting differential splicing of TGFBR1 exon 5 cause either Loeys-Dietz syndrome or multiple self-healing squamous epithelioma. Eur. J. Hum. Genet..

[8] Richards S (2015). Standards and guidelines for the interpretation of sequence variants: a joint consensus recommendation of the American College of Medical Genetics and Genomics and the Association for Molecular Pathology. Genet. Med..

[9] Akhurst RJ (2012). The paradoxical TGF-β vasculopathies. Nat. Genet..

[10] Li W (2014). Tgfbr2 disruption in postnatal smooth muscle impairs aortic wall homeostasis. J. Clin. Invest..

[11] Gallo EM (2014). Angiotensin II-dependent TGF-β signaling contributes to Loeys-Dietz syndrome vascular pathogenesis. J. Clin. Invest..

[12] Takeda Norifumi, Hara Hironori, Fujiwara Takayuki, Kanaya Tsubasa, Maemura Sonoko, Komuro Issei (2018). TGF-β Signaling-Related Genes and Thoracic Aortic Aneurysms and Dissections. International Journal of Molecular Sciences.

[13] van der Pluijm I (2016). Defective connective tissue remodeling in Smad3 mice leads to accelerated aneurysmal growth through disturbed downstream TGF-β signaling. EBio Med..

[14] Habashi JP (2006). Losartan, an AT1 antagonist, prevents aortic aneurysm in a mouse model of Marfan syndrome. Science.

